# Diet-Independent Remodeling of Cellular Membranes Precedes Seasonally Changing Body Temperature in a Hibernator

**DOI:** 10.1371/journal.pone.0018641

**Published:** 2011-04-13

**Authors:** Walter Arnold, Thomas Ruf, Fredy Frey-Roos, Ute Bruns

**Affiliations:** Research Institute of Wildlife Ecology, University of Veterinary Medicine, Vienna, Austria; Vanderbilt University, United States of America

## Abstract

Polyunsaturated fatty acids (PUFA) have a multitude of health effects. Their incorporation into membrane phospholipids (PL) is generally believed to depend directly on dietary influx. PL influence transmembrane protein activity and thus can compensate temperature effects; e.g. PL n-6 PUFA are thought to stabilize heart function at low body temperature (T_b_), whereas long chain (>C18) n-3 PUFA may boost oxidative capacity. We found substantial remodeling of membranes in free-living alpine marmots which was largely independent of direct dietary supply. Organ PL n-6 PUFA and n-6 to n-3 ratios were highest at onset and end of hibernation after rapid increases during a brief transitional period prior to hibernation. In contrast, longer chain PL n-3 PUFA content was low at end of summer but maximal at end of hibernation. After termination of hibernation in spring, these changes in PL composition were rapidly reversed. Our results demonstrate selective trafficking of PUFA within the body, probably governed by a circannual endogenous rhythm, as hibernating marmots were in winter burrows isolated for seven months from food and external cues signaling the approaching spring. High concentrations of PL n-6 PUFA throughout hibernation are in line with their hypothesized function of boosting SERCA 2a activity at low T_b_. Furthermore, we found increasing rate of rewarming from torpor during winter indicating increasing oxidative capacity that could be explained by the accumulation of long-chain PL n-3 PUFA. It may serve to minimize the time necessary for rewarming despite the increasing temperature range to be covered, because rewarming is a period of highest metabolic rate and hence production of reactive oxygen species. Considering the importance of PUFA for health our results may have important biomedical implications, as seasonal changes of T_b_ and associated remodeling of membranes are not restricted to hibernators but presumably common among endothermic organisms.

## Introduction

Cellular membranes contain variable amounts of essential polyunsaturated fatty acids (PUFA), generally believed to depend directly on dietary influx and tissue specific differences [1]–[8]. However, rapid and diet-independent adjustments of membrane composition in response to changing cell temperature are well known for ectotherms [9], [10]. This so-called “homeoviscous adaptation” is thought to maintain membrane integrity at lower temperatures. Endothermic organisms also show fluctuations in body temperature (T_b_) on a daily and seasonal basis, carried to the extreme by hibernators and daily heterotherms [11]–[13]. Hence, integration of PUFA into phospholipids (PL) in order to cope with lower cell temperature may be conserved in endotherms. Indeed, studies in the laboratory found such changes in hibernating mammals [14]–[16], and in deer mice exhibiting a higher propensity for daily torpor induced by short photoperiod [17].

However, there is accumulating evidence against an effect of membrane unsaturation *per se*, but instead for specific interactions of n-6 and n-3 PUFA with proteins, e.g. of oxidative pathways, ATPases, and ion channels [15], [18]–[21]. For instance, incorporation of n-6 PUFA into PL is a well known phenomenon of cold acclimation [22]. A high n-6 to n-3 ratio has been suggested to mitigate temperature (Arrhenius) effects on the activity of the sarcoplasmatic reticulum Ca^2+^-Mg^2+^ pump in cardiac myocytes (SERCA 2a) of hibernating animals thereby enabling lower deep torpor T_b_, and thus less fat consumption, without jeopardizing heart function [21]. On the other hand, long-chain n-3 PUFA like docosapentaenoic acid (DPA, C22:5 n-3) and docosahexaenoic acid (DHA, C22:6 n-3) are thought to act as “metabolic pace-makers” [23], although they enhance metabolic scope rather than basal metabolic rate [20], [24]. However, high concentrations of n-3 PUFA seem to be incompatible with a life at low T_b_ (reviewed in [21]).

**Figure 1:**
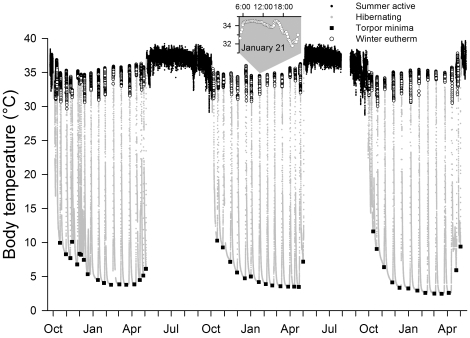
Analyzed body temperature parameters. Core body temperature (T_b_) course over three winters (inside a sealed hibernaculum) and two summer seasons in an adult marmot studied in its natural habitat as an example for T_b_ parameters and periods analyzed. Small black circles: T_b_ of summer active animal. Small grey circles: T_b_ during hibernation. Black squares: minimum T_b_ reached during a torpor bout. Large open circles: T_b_ during periods of winter euthermy defined as time between the end of continuous increase of T_b_ during rewarming and the onset of continuous decline of T_b_ into the next torpor bout (see inset for a magnified plot of a euthermy interval).

Therefore, we hypothesized that hibernators may undergo diet-independent adjustments of PUFA types and concentrations in PL, in preparation for seasonal changes in T_b_. Specifically, we expected an increase of n-6 PUFA in organ PL prior to and during hibernation but a decrease of n-3. To test these hypotheses in a single organism, we chose free-ranging alpine marmots (*Marmota marmota*) as model species. We considered this hibernator suitable because it shows profound changes of T_b_ throughout the year (Fig. 1), and relies entirely on fat reserves to fuel metabolism during hibernation and early spring [25]–[27], excluding any direct dietary influence on membrane PL composition during these periods. Furthermore, alpine marmots live in winter burrows for seven months each year, isolated from food and external cues signaling the approaching spring. Thus, changes occurring during hibernation would indicate endogenous control.

## Results

### Thermoregulation

**Figure 2:**
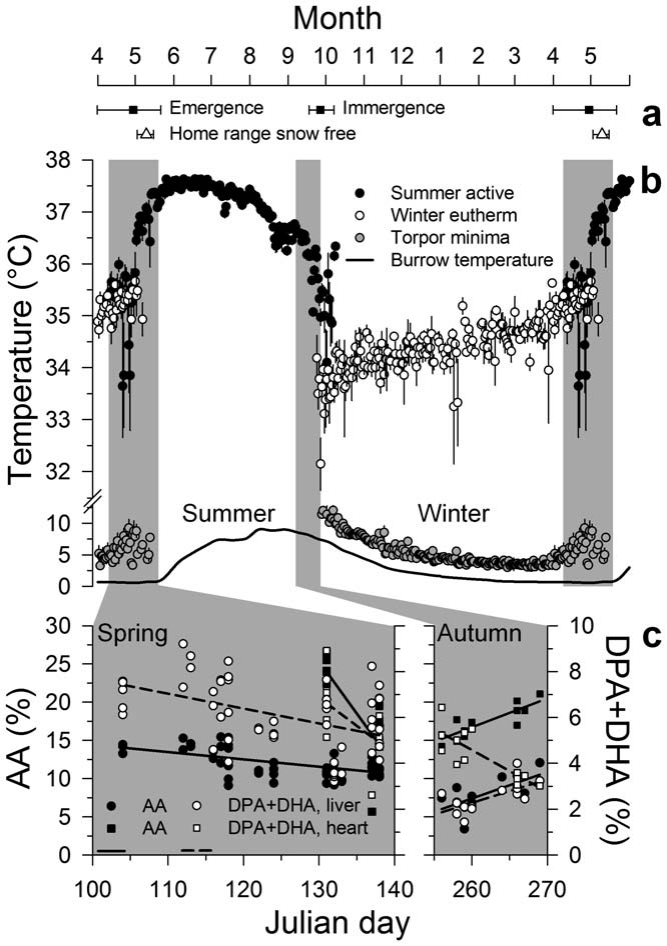
Seasonality of body temperature and membrane composition. **a** Means and ranges of dates of emergence from and immergence into hibernacula (black squares and horizontal lines) of marmots equipped with transmitters; mean and range of dates when snow cover had disappeared from approximately ¾ of the marmots' home ranges as an approximation of the onset of vegetation growth (open triangles and horizontal lines). **b** Body temperature (T_b_) of summer active (black circles), and winter euthermic (white circles) marmots, minimum T_b_ reached during bouts of torpor (grey circles) and burrow temperature (line). Data in **a** and **b** are double plotted for months April and May to ease visualization of the seasonal pattern. Symbols represent daily means, error bars (standard error of the mean, s.e.) reflect variation between individuals, missing error bars indicate s.e. smaller than symbol size. Burrow temperature is a line plot of daily means smoothed with a cubic spline. **c** Concentrations of long-chain n-6 (arachidonic acid (AA), C20:4 n-6, black symbols), and n-3 fatty acids (docosapentaenoic acid (DPA), C22:5 n-3, and docosahexaenoic acid (DHA), C22:6 n-3, white symbols) in phospholipids of liver (circles, dashed regression lines) and heart (squares, solid regression lines), of marmots shot during Spring and Autumn hunting periods (shaded sections). Data shown here are examples of several fatty acids that changed significantly (Fig. 4, Tab. 1).

We continuously measured T_b_ telemetrically in the abdominal cavity of 53 animals in their natural habitat, altogether 154 animal years. Daily mean T_b_ was highest in June and decreased during the rest of summer, at first slowly, but with a profound drop just prior to hibernation. T_b_ of hibernating animals approached the temperature in the burrow and followed its winter decline to a minimum of 1.8°C (Fig. 2b). Marmots, like all hibernators, regularly interrupted hibernation [25], [28] (Fig. 1) and returned to high T_b_ for on average 27.8±2.54 h (± standard error of the mean). Mean T_b_ during these periods of so-called “euthermy” (Fig. 1, inset graph) was 2.4°C below the daily mean T_b_ of summer active marmots, and still 0.7±0.05°C (F_(1,8896)_ = 168.0, p<0.001) below daily minimum T_b_ during summer. However, euthermic T_b_ increased continuously during winter, and rapidly increased to summer levels at the end of hibernation within about two to three weeks (Fig. 2b). Four exceptionally low spring values of daily mean T_b_ resulted from occasional short-term relapses of some animals into torpor after opening the winter burrow (Fig. 2b).

**Figure 3:**
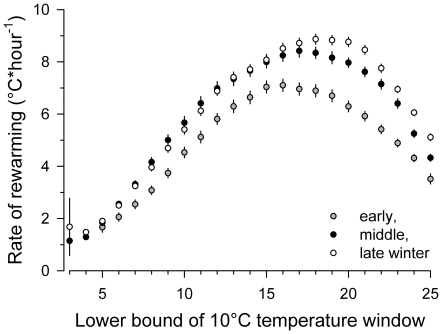
Rate of rewarming and its change during winter. Rate of rewarming from torpor to euthermy, calculated for 10°C temperature windows moving in 1°C steps from 3 to 35°C, during arousals in the early (begin to Dec 5), middle (Dec 6 to Feb 28), and late (Mar 1 to termination) third of the hibernation season. Values are means ± s.e. of moving 10°C averages. Error bars reflect variation between individuals; missing error bars indicate s.e. smaller than symbol size.

Rewarming from torpor was slow during the initial phase, reached highest rates from 18 to 28°C (mean 8.1±8.23°C*h^−1^) and decelerated when T_b_ approached euthermic levels. Interestingly, rewarming accelerated during winter (Fig. 3) with decreasing minimal deep torpor T_b_ (Fig. 2b). To some degree, this could be explained by decreasing body mass of winter fasting marmots (effect on logarithmic rate of rewarming: standardized partial regression coefficient −0.31, F_(1,783)_ = 43.3, p<0.001; body mass assumed to decrease linearly from immergence to emergence mass). However, independent of body mass, rewarming rate increased with decreasing initial T_b_ (effect of minimal T_b_ during the preceding bout of torpor on logarithmic rate of rewarming: standardized partial regression coefficient −0.24, F_(1,783)_ = 53.1, p<0.001).

### Remodeling of cell membranes

**Figure 4:**
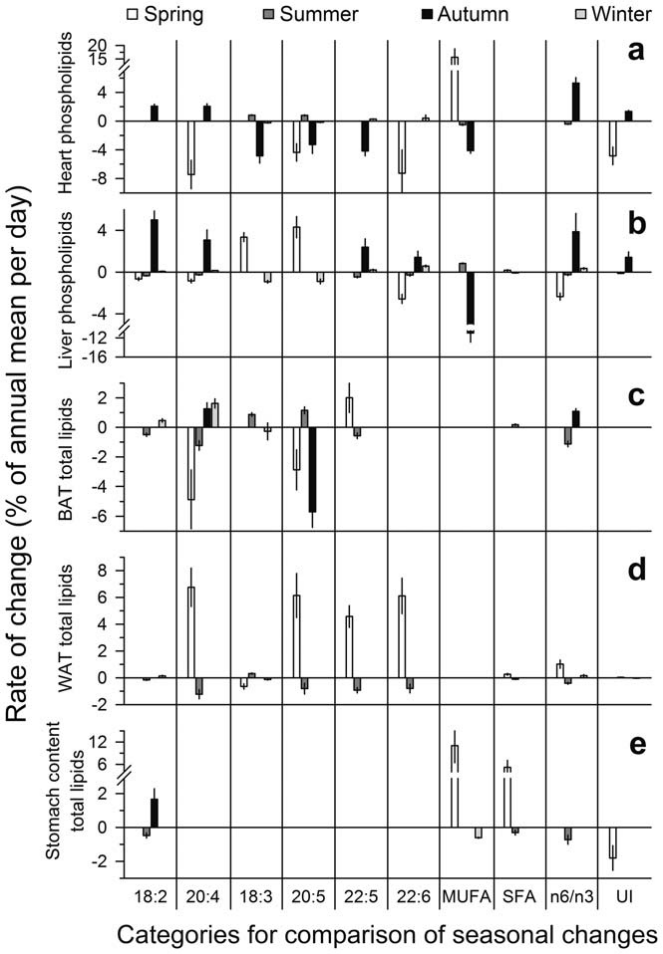
Rate of fatty acid changes. Rates of change per day (%) during the Spring and Autumn sampling period, and in between (Summer and Winter, respectively, see Fig. 2 for definition), of concentrations of various fatty acids in heart (a) and liver phospholipids (b), in total lipids of brown adipose tissue (c), peritoneal white adipose tissue (d), and stomach content (e, only ≤C18 fatty acids analyzed), and the respective changes in n-6 to n-3 ratios and unsaturation index (UI). To ease comparisons of variables with large differences in scale, plotted values are normalized to percentage deviation from the annual mean of the respective variable. Only variables that changed significantly (p<0.05) during the respective period are plotted.

The autumn drop in T_b_ was paralleled by a massive influx of linoleic acid (LA, C18:2 n-6) and arachidonic acid (AA, C20:4 n-6) into heart and liver membranes. These n-6 PUFA replaced monounsaturated fatty acids (MUFA, C16:1 n-7 and C18:1 n-9), and, in the heart, also n-3 PUFA. In sum, during only 14 days, PUFA of the n-6 series increased in heart PL from 37.65±0.63% to 49.30±0.82% (F_(1,11)_ = 97.9, p<0.001), and in liver PL from 14.32±1.73% to 32.13±1.87% (F_(1,11)_ = 34.3, p<0.001). As a result, the n6/n3 ratio more than doubled in both organs. These trends continued during winter in the liver but not in the heart (Fig. 2c, Fig. 4, Tab. 1).

**Table 1:** Seasonal changes of phospholipid composition in organs.

		Spring						Summer						Autumn						Winter					
Tissue		*Mean*	*s.e.*	*slope*	*s.e.*	*df*	*p*	*Mean*	*s.e.*	*slope*	*s.e.*	*df*	*p*	*Mean*	*s.e.*	*slope*	*s.e.*	*df*	*p*	*Mean*	*s.e.*	*slope*	*s.e.*	*df*	*p*
*Heart*	20:4 n-6	23.98	1.67	−1.367	0.364	1,9	0.005	14.41	1.89	+0.004	0.008	1,20	0.661	15.20	0.46	+0.377	0.066	1,11	<0.001	20.11	0.60	+0.017	0.009	1,20	0.061
	18:2 n-6	26.03	1.91	−0.406	0.416	1,9	0.355	23.18	2.16	−0.005	0.015	1,20	0.719	22.44	0.49	+0.519	0.070	1,11	<0.001	29.20	0.63	−0.004	0.010	1,20	0.168
	22:6 n-3	2.43	0.24	−0.115	0.052	1,9	0.053	1.63	0.27	−0.002	0.002	1,20	0.389	1.37	0.15	−0.039	0.021	1,11	0.091	0.86	0.19	+0.007	0.001	1,20	<0.001
	22:5 n-3	4.18	0.36	−0.313	0.079	1,9	0.129	3.26	0.41	+0.005	0.003	1,20	0.113	3.94	0.16	−0.144	0.023	1,11	<0.001	2.07	0.21	+0.009	0.002	1,20	<0.001
	20:5 n-3	1.67	0.13	−0.100	0.028	1,9	0.006	0.96	0.14	+0.018	0.002	1,20	<0.001	3.49	0.20	−0.075	0.029	1,11	0.024	2.51	0.26	−0.004	0.001	1,20	0.007
	18:3 n-3	1.80	0.13	+0.029	0.028	1,9	0.328	2.01	0.15	+0.031	0.002	1,20	<0.001	6.23	0.26	−0.181	0.037	1,11	<0.001	3.99	0.34	−0.009	0.002	1,20	<0.001
	MUFA	7.46	1.58	+1.732	0.343	1,9	<0.001	19.58	1.78	−0.055	0.012	1,20	<0.001	11.98	0.28	−0.456	0.041	1,11	<0.001	6.06	0.37	+0.006	0.008	1,20	0.443
	SFA	32.45	2.18	+0.359	0.474	1,9	0.468	34.96	2.45	+0.003	0.016	1,20	0.868	35.34	0.31	−0.002	0.044	1,11	0.969	35.32	0.40	−0.013	0.011	1,20	0.257
	n-6/n-3	5.04	0.35	−0.050	0.076	1,9	0.525	4.69	0.39	−0.017	0.003	1,20	<0.001	2.37	0.22	+0.220	0.032	1,11	<0.001	5.23	0.28	−0.001	0.002	1,20	0.695
	UI	214.28	10.43	−8.938	2.271	1,9	0.003	151.71	11.76	+0.131	0.079	1,20	0.112	169.79	1.82	+2.489	0.261	1,11	<0.001	202.15	2.35	+0.053	0.052	1,20	0.319
*Liver*	20:4 n-6	14.05	0.48	−0.096	0.021	1,52	<0.001	10.80	0.35	−0.029	0.005	1,63	<0.001	6.01	0.82	+0.347	0.111	1,11	0.010	10.52	0.89	+0.018	0.005	1,63	<0.001
	18:2 n-6	24.60	0.73	−0.134	0.031	1,52	<0.001	20.03	0.53	−0.071	0.008	1,63	<0.001	8.30	1.27	+1.023	0.172	1,11	<0.001	21.60	1.37	+0.015	0.008	1,63	0.053
	22:6 n-3	4.52	0.27	−0.070	0.012	1,52	<0.001	2.15	0.20	−0.008	0.003	1,63	0.008	0.90	0.12	+0.039	0.016	1,11	0.034	1.40	0.13	+0.016	0.003	1,63	<0.001
	22:5 n-3	2.91	0.19	+0.005	0.008	1,52	0.566	3.06	0.14	−0.013	0.002	1,63	<0.001	0.97	0.16	+0.064	0.021	1,11	0.012	1.80	0.17	+0.006	0.002	1,63	0.004
	20:5 n-3	−0.10	0.37	+0.067	0.016	1,52	<0.001	2.18	0.27	+0.003	0.004	1,63	0.395	2.73	0.36	−0.003	0.048	1,11	0.958	2.70	0.39	−0.014	0.004	1,63	<0.001
	18:3 n-3	0.86	0.56	+0.187	0.024	1,52	<0.001	7.21	0.41	+0.010	0.007	1,63	0.121	8.91	1.21	+0.033	0.163	1,11	0.843	9.34	1.30	−0.042	0.006	1,63	<0.001
	MUFA	15.24	0.63	−0.028	0.027	1,52	0.301	14.28	0.46	+0.141	0.009	1,63	<0.001	37.51	2.40	−1.850	0.325	1,11	<0.001	13.46	2.59	+0.009	0.009	1,63	0.303
	SFA	37.94	0.52	+0.069	0.223	1,52	0.004	40.27	0.39	−0.034	0.007	1,63	<0.001	34.66	1.46	+0.347	0.198	1,11	0.107	39.18	1.58	−0.006	0.006	1,63	0.323
	n-6/n-3	4.39	0.22	−0.065	0.009	1,52	<0.001	2.17	0.16	−0.007	0.002	1,63	0.005	1.03	0.36	+0.108	0.049	1,11	0.050	2.43	0.39	+0.010	0.002	1,63	<0.001
	UI	173.68	3.21	−0.225	0.138	1,52	0.110	166.03	2.37	−0.197	0.037	1,63	<0.001	133.49	6.29	+2.340	0.850	1,11	0.019	163.9	6.78	+0.049	0.034	1,63	0.160

Concentration of various fatty acids in heart and liver phospholipids (%), n-6/n-3 ratio, and unsaturation index (UI) at the beginning of the Spring, Summer, Autumn, and Winter periods defined in Fig. 2, and daily changes during these periods (“slope” with its s.e., degrees of freedom (df), and p-value). For details on how listed values were calculated, see Methods.

Long-chain n-3 PUFA were found in much lower concentrations than AA at any time of the year, particularly in the heart. However, DHA and DPA increased remarkably during winter in both organs (heart: from 2.93±0.35%, to 6.62±0.57%, F_(1,20)_ = 26.9, p<0.001; liver: from 3.20±0.28%, to 7.42±0.42%, F_(1,63)_ = 28.2, p<0.001), a process that in liver PL had already begun in autumn. Altogether, the autumn and winter changes led to a peak in the unsaturation index (UI, total number of double bonds per 100 acyl chains) at the end of hibernation (Fig. 2c, Fig. 4, Tab. 1).

In spring, PL composition of organs changed again, concurrent with the increase in euthermic T_b_. The total concentration of AA, DHA and DPA decreased over only 8 days in the heart from 30.59±1.98% to 19.30±2.23% (F_(1,9)_ = 14.0, p = 0.005), and in the liver over 35 days of sampling from 21.47±0.78% to 16.02±0.57% (F_(1,52)_ = 23.1, p<0.001). LA concentrations also decreased during spring. Long-chain PUFA were replaced predominantly by MUFA in the heart, but in the liver by α-linolenic acid (ALA C18:3 n-3), leading to significantly lower UI in the heart and n6/n3 ratio in the liver at the end of the spring sampling period. During summer, most of the spring trends continued, albeit at much lower rates (Fig. 2c, Fig. 4, Tab. 1).

### Polyunsaturated fatty acids in stomach contents and adipose tissue

MUFA cannot be converted to PUFA by vertebrates. ALA, the precursors of all longer chain n-3 PUFA, and LA, the precursor of AA, must be acquired in the diet as essential nutrients [23]. Nevertheless, the seasonal changes in heart and liver PL composition were largely independent of current dietary intake (cf. Tab. 1, Tab. 2). This is not surprising because food intake was low or absent for most of the year. Fill of the gut decreased during autumn until food intake ceased completely with the onset of hibernation (Tab. 3). When marmots first opened their burrows in spring, their home ranges were still covered by a thick snow pack, typically of one to three meters at the altitude of our study area (cf. [29]). Only with proceeding snow melt did the vegetation become gradually accessible (Fig. 2a), enabling a progressive increase in food intake after hibernation (effect of % snow cover in a home range on mass of gut fill in adult marmots: regression coefficient −2.64±0.49 g, F_(1,26)_ = 28.8, p<0.001, adj. R^2^ = 0.51). Further, the mean LA concentration in stomach contents during autumn, when concentrations of n-6 PUFA increased rapidly in organ PL, was even lower than during the spring period (14.38±0.86% vs. 18.11±0.80%; F_(1,21)_ = 10.0, p = 0.005), when n-6 PUFA were cleared from the organs.

**Table 2:** Seasonal changes of total lipid composition in stomach content.

	Spring						Summer						Autumn						Winter					
	*Mean*	*s.e.*	*slope*	*s.e.*	*df*	*p*	*Mean*	*s.e.*	*slope*	*s.e.*	*df*	*p*	*Mean*	*s.e.*	*slope*	*s.e.*	*df*	*p*	*Mean*	*s.e.*	*slope*	*s.e.*	*df*	*p*
18:2 n-6	18.17	1.19	−0.024	0.315	1,11	0.941	18.03	1.34	−0.079	0.024	1,19	0.004	12.62	0.93	+0.274	0.103	1,8	0.029	16.73	1.11	+0.013	0.015	1,19	0.422
18:3 n-3	64.00	2.97	−1.536	0.787	1,11	0.077	54.78	3.33	+0.118	0.059	1,19	0.060	62.84	1.99	−0.241	0.219	1,8	0.303	59.22	2.36	+0.042	0.037	1,19	0.271
MUFA	3.10	1.07	+0.679	0.283	1,11	0.035	7.17	1.20	+0.128	0.021	1,19	0.558	8.04	0.76	−0.049	0.084	1,8	0.579	7.32	0.90	−0.037	0.014	1,19	0.013
SFA	14.73	1.19	+0.882	0.314	1,11	0.017	20.02	1.33	−0.052	0.024	1,19	0.044	16.50	0.86	+0.016	0.094	1,8	0.873	16.73	1.01	−0.018	0.015	1,19	0.258
n-6/n-3	0.29	0.04	+0.009	0.010	1,11	0.412	0.34	0.04	−0.002	0.001	1,19	0.014	0.20	0.02	+0.005	0.003	1,8	0.064	0.29	0.03	0.000	0.001	1.19	0.921
UI	231.44	6.05	−3.978	1.603	1,11	0.031	207.57	6.79	+0.104	0.059	1,19	0.095	221.80	3.77	−0.224	0.415	1,8	0.605	218.44	4.47	+0.057	0.037	1,19	0.142

Concentration of various fatty acids in total lipids (%), n-6/n-3 ratio and unsaturation index (UI) of stomach content, at the beginning of the Spring, Summer, Autumn, and Winter period defined in Fig. 2, and daily changes during these periods (“slope” with its s.e., degrees of freedom (df), and p-value). For details on how listed values were calculated, see Methods.

**Table 3:** Seasonal changes of gut fill mass.

	Spring						Summer						Autumn						Winter					
*Gut section*	*Mean*	*s.e.*	*slope*	*s.e.*	*df*	*p*	*Mean*	*s.e.*	*slope*	*s.e.*	*df*	*p*	*Mean*	*s.e.*	*slope*	*s.e.*	*df*	*p*	*Mean*	*s.e.*	*slope*	*s.e.*	*df*	*p*
Stomach	−17.94	22.21	+4.373	0.942	1,31	<0.001	130.75	16.32	+2.800	0.525	1,42	<0.001	361.71	54.56	−25.240	7.373	1,11	0.006	33.59	58.87	−0.515	0.498	1,42	0.306
Small intestine	−9.48	7.79	+2.625	0.330	1,31	<0.001	79.77	5.72	+1.443	0.180	1,42	<0.001	198.85	18.43	−12.063	2.490	1,11	<0.001	42.03	19.89	−0.515	0.171	1,42	0.004
Caecum	−19.63	12.94	+4.039	0.549	1,31	<0.001	117.69	9.44	+1.217	0.241	1,42	<0.001	218.06	17.13	−9.905	2.315	1,11	0.001	89.29	18.48	−1.089	0.228	1,42	<0.001
Proximal colon	−6.12	7.16	+1.973	0.304	1,31	<0.001	60.96	5.26	+1.043	0.144	1,42	<0.001	147.0	12.30	−8.931	1.662	1,11	<0.001	30.89	13.27	−0.370	0.137	1,42	0.001
Distal colon	−1.16	6.79	+1.377	0.288	1,31	<0.001	45.64	4.99	+0.170	0.126	1,42	0.901	47.04	11.39	−0.827	1.539	1,11	0.602	36.29	12.29	−0.375	0.129	1,42	0.006
Whole gut	−54.33	43.15	+14.387	1.831	1,31	<0.001	434.81	31.70	+6.519	0.829	1,42	<0.001	972.65	64.44	−56.966	8.708	1,11	<0.001	232.09	69.53	−2.864	0.786	1,42	<0.001

Fill mass (g) of the alimentary tract of adult marmots at the beginning of the Spring, Summer, Autumn, and Winter period defined in Fig. 2, and daily changes during these periods (“slope” with its s.e., degrees of freedom (df), and p-value). For details on how listed values were calculated, see Methods.

Changes in organ PL were also largely independent from the fatty acid composition of white adipose tissue (WAT) (cf. Tab. 1, Tab. 4). During spring, the total concentration of AA, DPA and DHA in WAT increased from 0.20±0.32% to 1.32±0.23% (F_(1,48)_ = 6.0, p = 0.018). In contrast to WAT, total lipid composition of brown adipose tissue (BAT) resembled the fatty acid composition of heart and liver PL (about ¾ of changes in the same directions, cf. Tab. 1, Tab. 5). For instance, DPA and DHA increased in BAT from 0.99±0.41% at the onset of hibernation to 1.84±0.29% at emergence in spring (F_(1,33)_ = 3.2, p = 0.085, Tab. 5), but no such change occurred in WAT (F_(1,61)_ = 0.1, p = 0.711, Tab. 4); AA increased significantly during autumn and winter in BAT total lipids, like in organ PL (cf. Tab. 1, Tab. 5, Fig. 4), whereas it remained unchanged at negligibly low concentrations in WAT (Tab. 4, Fig. 4).

**Table 4:** Seasonal changes of total lipid composition in white adipose tissue.

		Spring						Summer						Autumn						Winter					
Tissue		*Mean*	*s.e.*	*slope*	*s.e.*	*df*	*p*	*Mean*	*s.e.*	*slope*	*s.e.*	*df*	*p*	*Mean*	*s.e.*	*slope*	*s.e.*	*df*	*p*	*Mean*	*s.e.*	*slope*	*s.e.*	*df*	*p*
*Subcutaneous*	20:4 n-6	−0.19	0.18	+0.037	0.008	1,188	<0.001	1.23	0.15	−0.007	0.001	1,215	<0.001	0.14	0.05	+0.004	0.006	1,27	0.467	0.22	0.07	−0.002	0.002	1,215	0.289
*WAT*	18:2 n-6	9.70	0.40	+0.024	0.017	1,188	0.171	10.64	0.33	−0.015	0.004	1,215	<0.001	8.13	0.44	−0.062	0.046	1,27	0.181	6.94	0.55	+0.014	0.005	1,215	0.003
	22:6 n-3	−0.04	0.06	+0.012	0.003	1,188	<0.001	0.41	0.05	−0.002	0.001	1,215	0.016	0.16	0.05	−0.010	0.005	1,27	0.072	−0.03	0.07	−0.000	0.000	1,215	0.927
	22:5 n-3	0.09	0.15	+0.037	0.007	1,188	<0.001	1.55	0.13	−0.008	0.001	1,215	<0.001	0.30	0.04	−0.005	0.004	1,27	0.240	0.21	0.05	−0.001	0.002	1,215	0.727
	20:5 n-3	−0.06	0.08	+0.014	0.004	1,188	<0.001	0.47	0.07	−0.002	0.001	1,215	0.046	0.18	0.03	−0.004	0.003	1,27	0.193	0.10	0.04	−0.001	0.001	1,215	0.389
	18:3 n-3	19.41	0.83	−0.115	0.036	1,188	0.002	14.93	0.69	+0.056	0.009	1,215	<0.001	24.24	0.83	−0.025	0.087	1,27	0.772	23.76	1.05	−0.022	0.001	1,215	0.020
	MUFA	51.37	0.90	−0.646	0.039	1,188	0.100	48.85	0.75	−0.001	0.001	1,215	0.796	48.62	1.13	+0.024	0.118	1,27	0.841	49.07	1.43	−0.011	0.010	1,215	0.264
	SFA	19.75	0.33	+0.054	0.014	1,188	<0.001	21.85	0.28	−0.223	0.037	1,215	<0.001	18.17	0.41	+0.081	0.042	1,27	0.068	19.72	0.52	+0.000	0.004	1,215	0.963
	n-6/n-3	0.49	0.04	+0.006	0.002	1,188	0.001	0.72	0.03	−0.002	0.001	1,215	<0.001	0.33	0.02	−0.002	0.002	1,27	0.430	0.31	0.03	+0.001	0.001	1,215	0.035
	UI	128.11	1.64	+0.109	0.071	1,188	0.126	132.36	1.36	+0.055	0.018	1,215	0.002	141.49	1.76	−0.264	0.183	1,27	0.161	136.47	2.23	−0.042	0.018	1,215	0.024
*Peritoneal*	20:4 n-6	0.01	0.10	+0.001	0.004	1,51	0.049	0.31	0.08	+0.002	0.002	1,64	0.252	0.15	0.02	+0.005	0.004	1,13	0.217	0.21	0.03	−0.002	0.002	1,64	0.284
*WAT*	18:2 n-6	9.91	0.64	+0.002	0.028	1,51	0.940	9.98	0.48	−0.028	0.016	1,64	0.017	7.64	0.65	−0.126	0.094	1,13	0.202	6.00	0.83	+0.039	0.012	1,64	0.002
	22:6 n-3	−0.02	0.06	+0.006	0.003	1,51	0.046	0.16	0.05	−0.002	0.001	1,64	0.101	0.02	0.02	+0.002	0.004	1,13	0.625	0.04	0.03	−0.001	0.001	1,64	0.568
	22:5 n-3	0.17	0.09	+0.012	0.004	1,51	0.006	0.56	0.07	−0.003	0.002	1,64	0.048	0.30	0.03	−0.001	0.004	1,13	0.021	0.18	0.03	−0.001	0.002	1,64	0.969
	20:5 n-3	0.09	0.05	+0.001	0.002	1,51	0.582	0.12	0.03	+0.001	0.001	1,64	0.852	0.14	0.01	−0.005	0.001	1,13	0.005	0.07	0.01	+0.001	0.001	1,64	0.867
	18:3 n-3	24.61	1.02	−0.206	0.044	1,51	<0.001	17.60	0.76	+0.084	0.018	1,64	<0.001	24.49	0.95	+0.055	0.139	1,13	0.700	25.20	1.22	−0.006	0.194	1,64	0.765
	MUFA	45.86	0.98	+0.145	0.043	1,51	0.001	50.77	0.73	−0.046	0.019	1,64	0.021	47.00	1.59	+0.109	0.231	1,13	0.645	48.42	2.04	−0.026	0.021	1,64	0.217
	SFA	19.37	0.35	+0.031	0.015	1,51	0.050	20.41	0.26	−0.002	0.007	1,64	0.786	20.26	0.50	−0.030	0.073	1,13	0.692	19.88	0.64	−0.005	0.007	1,64	0.482
	n-6/n-3	0.37	0.06	+0.006	0.003	1,51	0.012	0.59	0.04	−0.003	0.001	1,64	<0.001	0.31	0.02	−0.005	0.003	1,13	0.107	0.24	0.03	+0.001	0.001	1,64	0.208
	UI	138.50	2.05	−0.340	0.089	1,51	<0.001	126.93	1.54	+0.113	0.038	1,64	0.004	136.25	2.49	−0.020	0.362	1,13	0.956	135.99	3.19	+0.025	0.041	1,64	0.537
*Cardial*	20:4 n-6	0.13	0.07	+0.006	0.003	1,50	0.080	0.33	0.05	−0.001	0.001	1,63	0.323	0.22	0.06	0.000	0.008	1,13	0.995	0.22	0.08	−0.000	0.001	1,63	0.526
*WAT*	18:2 n-6	11.06	0.06	0.000	0.002	1,50	0.996	11.06	0.42	−0.015	0.005	1,63	0.004	8.56	0.57	−0.099	0.083	1,13	0.254	7.27	0.73	+0.019	0.005	1,63	<0.001
	22:6 n-3	−0.05	0.14	+0.010	0.006	1,50	0.098	0.28	0.10	−0.002	0.001	1,63	0.185	0.03	0.04	+0.002	0.005	1,13	0.701	0.06	0.05	−0.001	0.001	1,63	0.648
	22:5 n-3	0.35	0.09	+0.007	0.004	1,50	0.086	0.58	0.07	−0.001	0.001	1,63	0.236	0.43	0.04	−0.018	0.006	1,13	0.015	0.20	0.06	−0.001	0.001	1,63	0.361
	20:5 n-3	0.13	0.02	−0.001	0.001	1,50	0.426	0.10	0.01	+0.001	0.000	1,63	0.007	0.19	0.03	−0.009	0.004	1,13	0.041	0.07	0.04	+0.000	0.000	1,63	0.157
	18:3 n-3	22.72	1.06	−0.106	0.046	1,50	0.025	19.12	0.78	+0.034	0.009	1,63	<0.001	24.72	0.85	−0.100	0.123	1,13	0.431	23.42	1.09	−0.004	0.010	1,63	0.722
	MUFA	44.01	1.08	+0.053	0.046	1,50	0.256	45.82	0.80	−0.011	0.010	1,63	0.251	43.94	1.29	+0.141	0.187	1,13	0.463	45.77	1.65	−0.009	0.011	1,63	0.404
	SFA	20.70	0.36	+0.021	0.015	1,50	0.187	21.40	0.26	0.000	0.000	1,63	0.256	21.03	0.33	+0.058	0.048	1,13	0.256	21.77	0.42	−0.005	0.003	1,63	0.116
	n-6/n-3	0.48	0.05	+0.003	0.002	1,50	0.101	0.60	0.04	−0.002	0.000	1,63	<0.001	0.35	0.02	−0.002	0.003	1,13	0.496	0.32	0.03	+0.001	0.000	1,63	0.070
	UI	136.88	2.12	−0.151	0.091	1,51	0.105	131.74	1.57	+0.046	0.019	1,63	0.017	139.39	2.12	−0.478	0.308	1,13	0.144	133.17	2.71	+0.019	0.020	1,63	0.363
*Renal*	20:4 n-6	−0.02	0.14	+0.011	0.006	1,51	0.077	0.36	0.10	−0.001	0.001	1,64	0.300	0.16	0.03	+0.004	0.004	1,13	0.288	0.21	0.03	−0.001	0.000	1,64	0.368
*WAT*	18:2 n-6	10.01	0.71	−0.002	0.030	1,51	0.942	9.93	0.51	−0.016	0.006	1,64	0.012	7.36	0.53	−0.102	0.077	1,13	0.208	6.04	0.68	+0.020	0.007	1,64	0.003
	22:6 n-3	−0.06	0.12	+0.008	0.005	1,51	0.096	0.23	0.08	−0.001	0.001	1,64	0.173	0.01	0.02	+0.002	0.003	1,13	0.404	0.04	0.03	−0.001	0.001	1,64	0.614
	22:5 n-3	0.21	0.13	+0.010	0.006	1,51	0.083	0.55	0.09	−0.002	0.001	1,64	0.147	0.29	0.02	−0.008	0.002	1,13	0.004	0.18	0.02	+0.000	0.001	1,64	0.913
	20:5 n-3	0.08	0.05	−0.001	0.002	1,51	0.617	0.11	0.03	+0.000	0.000	1,64	0.846	0.13	0.02	−0.003	0.002	1,13	0.152	0.08	0.02	0.000	0.000	1,64	0.981
	18:3 n-3	23.52	1.01	−0.170	0.043	1,51	<0.001	17.75	0.71	+0.038	0.009	1,64	<0.001	24.08	0.84	+0.078	0.122	1,13	0.537	25.09	1.08	−0.008	0.009	1,64	0.399
	MUFA	46.09	1.29	+0.116	0.055	1,51	0.040	50.03	0.92	−0.014	0.011	1,64	0.222	47.74	1.34	+0.005	0.195	1,13	0.978	47.81	1.72	−0.009	0.012	1,64	0.481
	SFA	20.19	0.40	+0.019	0.017	1,51	0.262	20.85	0.28	−0.004	0.004	1,64	0.294	20.24	0.43	+0.024	0.062	1,13	0.708	20.55	0.55	−0.002	0.004	1,64	0.641
	n-6/n-3	0.41	0.05	+0.005	0.002	1,51	0.031	0.57	0.04	−0.002	0.000	1,64	<0.001	0.31	0.02	−0.005	0.002	1,13	0.072	0.25	0.02	+0.001	0.000	1,64	0.073
	UI	137.68	2.45	−0.249	0.105	1,51	0.021	129.22	1.74	+0.050	0.021	1,64	0.020	137.47	2.14	+0.008	0.310	1,13	0.980	137.57	2.74	−0.001	0.023	1,64	0.981

Concentration of various fatty acids in total lipids (%), n-6/n-3 ratio and unsaturation index (UI) of various depots of white adipose tissue (WAT), at the beginning of the Spring, Summer, Autumn, and Winter period defined in Fig. 2, and daily changes during these periods (“slope” with its s.e., degrees of freedom (df), and p-value). For details on how listed values were calculated, see Methods.

**Table 5:** Seasonal changes of total lipid composition in brown adipose tissue.

	Spring						Summer						Autumn						Winter					
	*Mean*	*s.e.*	*slope*	*s.e.*	*df*	*p*	*Mean*	*s.e.*	*slope*	*s.e.*	*df*	*p*	*Mean*	*s.e.*	*slope*	*s.e.*	*df*	*p*	*Mean*	*s.e.*	*slope*	*s.e.*	*df*	*p*
20:4 n-6	8.86	1.22	−0.206	0.084	1,21	0.023	4.74	0.78	−0.052	0.014	1,33	<0.001	0.81	0.14	+0.053	0.020	1,12	0.018	1.50	0.17	+0.069	0.013	1,33	<0.001
18:2 n-6	19.26	1.39	−0.184	0.095	1,21	0.066	15.57	0.88	−0.072	0.017	1,33	<0.001	10.11	0.77	+0.140	0.109	1,12	0.224	11.92	0.96	+0.069	0.017	1,33	<0.001
22:6 n-3	0.70	0.12	−0.009	0.009	1,21	0.320	0.52	0.08	−0.001	0.004	1,33	0.800	0.45	0.35	−0.014	0.050	1,12	0.787	0.27	0.44	+0.004	0.004	1,33	0.282
22:5 n-3	1.14	0.19	+0.026	0.013	1,21	0.058	1.65	0.12	−0.007	0.002	1,33	0.004	1.10	0.11	−0.029	0.016	1,12	0.101	0.73	0.14	+0.004	0.002	1,33	0.104
20:5 n-3	0.73	0.12	−0.017	0.008	1,21	0.047	0.39	0.07	+0.007	0.001	1,33	<0.001	0.91	0.04	−0.034	0.006	1,12	<0.001	0.47	0.06	+0.002	0.001	1,33	0.080
18:3 n-3	14.63	1.75	−0.030	0.120	1,21	0.807	14.03	1.11	+0.165	0.024	1,33	<0.001	26.45	1.42	−0.013	0.065	1,12	0.949	26.28	1.77	−0.109	0.024	1,33	<0.001
MUFA	33.14	3.41	+0.416	0.234	1,21	0.091	41.45	2.17	−0.080	0.045	1,33	0.081	35.42	2.37	−0.015	0.336	1,12	0.966	35.24	2.96	−0.020	0.044	1,33	0.657
SFA	21.54	0.59	+0.005	0.041	1,21	0.905	21.64	0.38	+0.041	0.012	1,33	0.002	24.75	1.01	−0.089	0.143	1,12	0.544	23.58	1.26	−0.019	0.012	1,33	0.111
n-6/n-3	1.69	0.17	−0.022	0.012	1,21	0.082	1.26	0.11	−0.012	0.002	1,33	<0.001	0.38	0.03	+0.008	0.005	1,12	0.092	0.49	0.04	+0.012	0.002	1,33	<0.001
UI	160.22	7.71	−0.867	0.529	1,21	0.116	142.89	4.89	+0.059	0.091	1,33	0.524	147.32	2.74	+0.081	0.389	1,12	0.838	148.37	3.43	+0.111	0.089	1,33	0.219

Concentration of various fatty acids in total lipids (%), n-6/n-3 ratio and unsaturation index (UI) of brown adipose tissue, at the beginning of the Spring, Summer, Autumn, and Winter period defined in Fig. 2, and daily changes during these periods (“slope” with its s.e., degrees of freedom (df), and p-value). For details on how listed values were calculated, see Methods.

## Discussion

### Function of seasonal transitions

If a membrane rich in n-6 PUFA is necessary for maintaining heart function in hibernating mammals [21], it should be available when an animal undergoes the first bout of torpor. Indeed, the most remarkable increase in the proportion of PL n-6 PUFA occurred during a short pre-hibernation transition phase. Furthermore, this increase as well as total concentration was highest in the heart and n-6 concentration and n-6/n-3 ratio were highest immediately before and after hibernation. It is still unknown why an n-6 environment apparently stimulates SERCA 2a activity and stabilizes heart function at low T_b_. The explanation may lie in the coupling between the hydrophobic core of the lipid bilayer and the hydrophobic part of a membrane-spanning protein [30]. Any conformational change of a protein involving variation in its cross-sectional area causes local bilayer deformation with associated changes of the lateral pressure profile. Distribution of lateral stress within the hydrophobic core depends on the degree of unsaturation and alters the equilibrium constant for the protein conformational states. Even small changes in membrane composition, e.g. replacement of n-3 with n-6 PUFA, can lead to shifts in local pressure in the magnitude of hundreds of bars [31]–[33].

The second striking change during autumn and winter in organ PL composition was the accumulation of AA, DPA and DHA indicating chain-elongation and further unsaturation of precursors. Considering the similarity of seasonal changes found in organ PL and BAT total lipids, which is in sharp contrast to WAT total lipids, it seems reasonable to assume that changes in BAT-PL and not in BAT-triglycerides are responsible for this resemblance. Accumulation of DPA and DHA in PL may well explain the simultaneous increase in accelerated rewarming (which we assume indicates an increase in thermogenic capacity) and euthermic T_b_. DPA and DHA are well known to increase the activity of Krebs cycle and β-oxidation enzymes (reviewed in [20]). In addition, non-shivering thermogenesis could also be improved if uncoupling protein is not a proton channel but a fatty acid anion carrier [34]. In this case transport of protonated fatty acids across the inner mitochondrial membrane is rate limiting. It has been suggested that this transport occurs by flip-flop, known to be fastest in highly permeable DHA-rich membranes [35]. The function of increasing thermogenic capacity during winter may lie in keeping rewarming times short. Rapid rewarming time could minimize the total amount of detrimental reactive oxygen species (ROS) produced, despite the extraordinarily high metabolic rate during rewarming [27], [36], [37].

However, periods of high peroxidation risk of PUFA are short during hibernation [37] and antioxidant defense is upregulated [38]–[40]. The situation is presumably different in spring and early summer when the energetically most expensive tasks in a marmot's life take place: reproduction, competition for territory ownership, growth and fattening [41]. These tasks require continuously high metabolic rate, possibly rendering highly unsaturated membranes too prone to peroxidation. Furthermore, maintaining sufficient levels of antioxidant defense could be energetically too expensive in light of the high energy allocation towards competing tasks. Thus, a shift towards higher T_b_ allowing clearance of PUFA from PL but maintaining high aerobic power seems to be a better option [36].

### Source of membrane lipids

The LA incorporated into organ PL most likely originated predominantly from the huge amount of WAT built up during summer fattening (estimated body mass of adult marmots at emergence from hibernation 2915±87 g, n = 172, at immergence 4466±219 g, n = 29; mean concentration of LA across four WAT depots sampled at end of summer 7.84±0.58%, Tab. 4). However, preferential release of LA during lipolysis as an explanation for its increase in organ PL can be excluded. The concentration of LA in WAT increased during winter by 3.69±1.18% (regression of means of all WAT depots sampled, F_(1,61)_ = 9.8, p = 0.002) indicating its selective retention [42]. This phenomenon is common in hibernators [43], [44], and was expected because more unsaturated PUFA like ALA are preferentially mobilized during lipolysis [45], [46]. Therefore, if organ PL simply reflected concentrations of FA released from WAT into the blood, one would expect that - contrary to our results - more unsaturated PUFA, rather than LA, would show largest increases in organs. In conclusion, our findings implicate preferential incorporation of n-6 PUFA into organ PL or the existence of fatty acid specific trafficking between organs and WAT, by, to our knowledge, unknown means. Candidates are fatty acid transport proteins, suspected to bind specifically to PUFA [47], [48]. Reversal of membrane composition to the summer state during spring may have occurred by similar means, because disappearance of PUFA from organ PL occurred simultaneously to their increase in WAT. However, the changes in spring could have also emerged through metabolization of organ PL-PUFA and continued depletion of triglyceride stores after termination of hibernation causing a relative increase of the proportion of PL and hence long-chain PUFA in the total lipid pool of WAT.

### Conclusion

Our results indicate that seasonal acclimation is much more comprehensive in endotherms than recognized up to date. It comprises major and surprisingly fast changes in membrane composition. Chain elongation and further desaturation of LA and ALA, well known mechanism of temperature acclimation in ectotherms, seem to be conserved in mammals. Other unexpected findings are the selective transfer of LA from WAT into organ PL in preparation for winter and removal of PUFA from PL when returning to a life at high T_b_. Furthermore, the apparent lack of a direct influence of diet, and the fact that marmots spent 6–7 months in winter burrows, completely isolated from external cues signaling the approaching spring, suggest a large degree of endogenous control of these processes.

There is growing evidence that hibernation is simply the most extreme expression of hypometabolism, which appears to be a ubiquitous feature of endotherms to anticipate and cope with temporary cold and food shortage [13]. Even humans have a lower T_b_ during winter [49]–[51] and maximal aerobic power during summer [52]. Seasonal remodeling of membranes may be similarly ubiquitous (e.g. [1], [53]). Considering the importance of PUFA for health [5]–[8], [54], a better understanding of the functions and mechanism of membrane remodeling is fundamental for human medicine. For instance, it may well be that the profound late winter peak of cardiovascular mortality [55], [56] is due to higher n-6/n-3 ratios in cardiac myocytes at this time [8], reflecting a conserved pattern of seasonal membrane acclimation.

## Materials and Methods

### Ethics Statement

Treatment of marmots in this study was approved by the cantonal veterinary office of Grisons, Chur, Switzerland, no. 5/1997.

### Field study

We studied over three years a population of free-living alpine marmots in the Avers-Bregalga valley in the Swiss Canton Grisons (46°26′N, 9°34′E, 1600–2400 m above sea level). We trapped and marked 300 marmots from 32 social groups (from 20 groups all members) individually with TROVAN® (Trovan, Ltd., Douglas, UK) transponders, a permanent tattoo, and fur dye for identification from a distance. Trapped marmots were weighed to the nearest 50 g with a hand-held spring balance. Composition of social groups and their home range sizes were determined by trapping and observing. Except one individual hibernating alone during one winter after natal dispersal, group members always hibernated together in a single burrow [25], [26], [41]. Onset of hibernation was assumed for a social group when no marmot was seen above ground for at least five days in a group's home range or when entrances to the group's hibernaculum were found to be plugged with soil from inside. Termination of hibernation was assumed for a group when the first marmot emerged from the hibernaculum in spring. Patterns of snow melt were determined by inspecting home ranges every other day and estimating % of snow coverage.

### Measurement of body and burrow temperature

T_b_ was measured telemetrically in a subset of 53 marked animals living and hibernating in 13 social groups of 2 to 10 individuals (mean = 3.2, SD = 1.96, n = 32 group years). Altogether, we obtained 154 marmot years of T_b_ data. In 22 of the total of 32 hibernation groups studied over 3 winters were all members equipped with transmitters. Only one female produced a litter during our study but these juveniles died before their first hibernation. We included this female in our analysis because omitting these T_b_ data did not significantly change our results. Burrow temperatures were also measured telemetrically in 20 different hibernacula as described elsewhere [57]. We used self-constructed radio transmitters (accuracy ±0.1°C, mass 40 g for adults, 22 g for juveniles) with a temperature-dependent pulse rate calibrated in the range of 2 to 38°C at five equidistant intervals before usage. Each transmitter operated with a unique frequency between 148 to 149 MHz.

Transmitters were implanted into the abdominal cavity under anesthesia with a mixture of 20 mg/kg zolazepam/tiletamin (Zoletil®) and 10 mg/kg xylazine (Rompun®). Implantations were conducted at the end of August or beginning of September to ensure at least three weeks for wound healing prior to hibernation. Animals were released at capture locations at least 24 h after surgery and after a final examination by a veterinarian.

Telemetry data were recorded and stored automatically in a cabin located in the centre of the study area and transferred in larger data packages to Vienna via modem connection. Three receivers were switched from channel to channel by computers resulting in T_b_ recordings for individual animals approximately every 15 minutes. After each switch to a new channel, receiver frequency was automatically fine tuned in order to improve the signal to noise ratio, and signals were discriminated from noise with an algorithm based on detection of periodicity.

### Tissue samples

We sampled tissues during spring and autumn (Fig. 2, shaded sections) from 151 trapped and 68 shot marmots. Animals were killed in a population control program with a single neck or head shot by game wardens from the Wildlife and Fishery Department of the Swiss Canton Grisons. For trapped marmots, approximately 100 mg WAT was obtained surgically from the inguinal subcutaneous fat depot under anesthesia as described above. Shot marmots were dissected within 20 minutes and about 2 g of heart, liver, axillary BAT, peritoneal, perirenal, pericardial, and inguinal subcutaneous WAT, and stomach content were put in 2 ml polyethylene vials, flooded with nitrogen to prevent oxidation, snap-frozen in dry ice and stored at −80°C until analysis. The stomach content sample was taken from a larger (>50 g) thoroughly mixed quantity of stomach content. During one transport of samples from Switzerland to Vienna, we ran out of dry ice due an unexpected delay at the border. As a result, we lost heart samples from spring and could therefore analyze changes after hibernation in heart PL only during a smaller time window.

### Biochemical analyses

Triglyceride fatty acids comprise >95% of total lipids in rodent WAT [58]. Therefore, we did not separate triglycerides and PL prior to analysis in samples from WAT and BAT, but analyzed total lipids in all adipose tissues. In stomach contents we also analyzed total lipids. Organ PL were isolated using thin-layer chromatography [59] and transesterified thereafter with a one-step method also used for adipose tissue lipids [60], [61]. Fatty acids were identified by gas-liquid chromatography using a Perkin-Elmer FID AutoSystem XL autosampler chromatograph equipped with a 30 m×0.25 mm×0.25 µm HP INNOWax capillary column, using the following parameters: injector 240°C, column 130–180°C at 4°C/min, 180–200°C at 3°C/min, 200–240°C at 15°C/min, 240°C for 8 min. The relative fatty acid composition was quantified using external fatty acid methyl ester standards (Supelco) run after every 20 samples and Turbochrom 4.1 software (Perkin Elmer). Concentrations of single fatty acids were calculated as mass % of total identified peaks of fatty acids of chain length 14 to 22. In stomach contents, only lipids with chain lengths ≤C18 were analyzed because longer fatty acids were not detectable or only present at negligible concentrations. Percentages therefore refer for stomach contents to mass % of total identified peaks of fatty acids of chain length 14 to 18.

### Data analyses

Data analyses were carried out using the statistical package R [62]. Values presented in Tables 1, 2, 3, 4, 5 and Fig. 4 are calculated from regressions on data obtained during the two sampling periods after and before hibernation (Spring and Autumn respectively, shaded sections in Fig. 2). Intercepts of these regressions represent the estimated mean at the beginning of these periods, their slopes the average change per day. By reversing the time scale of these regressions, we estimated the intercepts at the end of these periods, representing the estimated means at the beginning of the Summer and Winter period (Fig. 2). Changes per day during Summer and Winter were calculated by dividing the differences between the intercepts at begin and end of these periods with the number of days between the last day of Spring and first day of Autumn, and the last day of Autumn and first day of Spring, respectively. Significance of changes per day during Summer and Winter was tested by comparing intercepts of Spring and Autumn regressions with one time scale reversed. For instance, a significant change during Winter was identified when the intercepts of the regression of Autumn samples against time with last day active as day 1 and Spring samples with first day active as day 1 differed with p<0.05. To avoid pseudoreplication, we analyzed temperatures sampled repeatedly from the same individuals in linear mixed effect models with a random effect “Individual” [63]. Violation of assumptions to be met for parametric testing was identified by inspecting residuals from statistical models and eliminated, if necessary, by appropriate transformation of data.
